# Contact-force-sensing-based radiofrequency catheter ablation in paroxysmal supraventricular tachycardias: A randomized controlled trial

**DOI:** 10.1016/j.hroo.2026.04.007

**Published:** 2026-04-15

**Authors:** Nawin L. Ramdat Misier, Tamas Geczy, Sip A. Wijchers, Rohit E. Bhagwandien, Mark Hoogendijk, Bakhtawar Khan Mahmoodi, Tamas Szili-Torok, Sing-Chien Yap

**Affiliations:** 1 Department of Cardiology, Thorax Center, Cardiovascular Institute, Erasmus MC, Rotterdam, The Netherlands; 2 Department of Cardiology, Cardiovascular Center Bad Bevensen, Bad Bevensen, Germany; 3 Department of Internal Medicine, Cardiology Center, University of Szeged, Szeged, Hungary

**Keywords:** Ablation, Atrioventricular nodal reentrant tachycardia, Atrioventricular reentrant tachycardia, Contact force, Supraventricular tachycardia, WPW

## Abstract

**Background:**

Although contact-force (CF) technology has gained traction in atrial fibrillation ablation, limited data exist regarding its utility in supraventricular tachycardia (SVT) ablation.

**Objective:**

This study aimed to evaluate whether CF-sensing catheter ablation offers procedural advantages over conventional ablation for treatment of SVT by comparing the number of radiofrequency (RF) applications and assessing safety, efficacy, and long-term outcomes.

**Method:**

In this randomized controlled trial, patients with SVT (atrioventricular nodal reentrant tachycardia/atrioventricular reentrant tachycardia) were assigned to either CF-sensing or conventional ablation. The primary end point was the total number of RF applications. Secondary endpoints included RF time, fluoroscopy time, procedural duration, acute and long-term success, and adverse events. Follow-up included electrocardiograms at 3 and 12 months, with Holter monitoring if symptoms recurred.

**Results:**

A total of 107 patients with SVT (mean age 49.4 ± 14.7 years; 47.7% male) were assigned to CF-sensing (n = 54) or conventional ablation (n = 53). Acute procedural success was achieved in 98.1% of patients in both arms. The median number of RF applications did not differ significantly between CF (3.5 [1.8–8.3]) and control groups (4.0 [2.0–11.0]; *P* = .43). There were no significant differences in RF time, fluoroscopy time, or procedural duration (*P* > .05). 12-month single-procedure success was 94.4% in the CF group vs 98.1% in the control group (*P* = .61). Adverse event rates were similar (0% CF vs 5.7% control; *P* = .12).

**Conclusion:**

CF-sensing catheter ablation did not reduce the number of applications or improve procedural metrics in SVT ablation. Both CF and conventional ablation demonstrated high efficacy and comparable safety profiles.

**Clinical trial registration:**

NCT04078685.

## Graphical abstract

**Figure:**
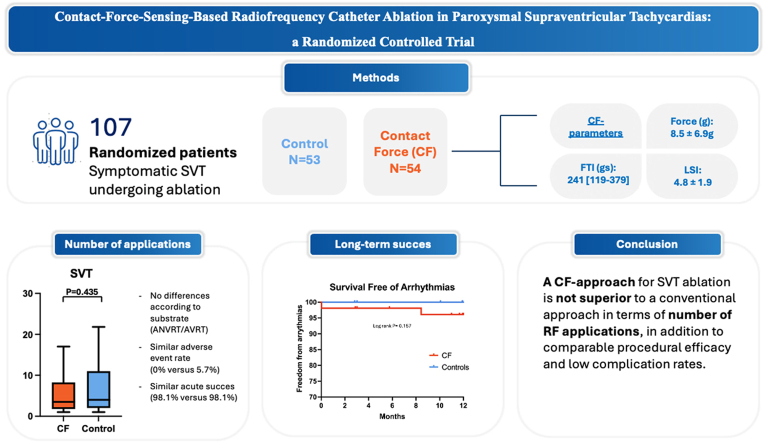

**Key Findings**

▪ The use of contact-force data during supraventricular tachycardia ablation did not result in a significant reduction in the number of radiofrequency applications, regardless of the arrhythmic substrate.
▪ Procedural efficacy and safety outcomes were similar between contact force–guided and conventional ablation strategies.
▪ The high success rates in both arms suggest a procedural performance ceiling that may not be improved by contact-force data alone.

## Introduction

Catheter ablation is the recommended treatment for patients with paroxysmal supraventricular tachycardia (SVT), particularly those who are symptomatic and either intolerant or refractory to antiarrhythmic medication.^1^ Multicenter studies focusing on both atrioventricular nodal reentrant tachycardia (AVNRT) and accessory pathway–mediated atrioventricular reentrant tachycardia (AVRT) have demonstrated high acute success rates with low complication rates.^2^, ^3^, ^4^, ^5^ However, (early) recurrences during follow-up remain a significant clinical challenge in selected patients, with recurrence rates ranging from 5.6% to 9.9%.^3^^,^^4^^,^^6^

Acute ablation failure and early recurrence have been attributed to suboptimal catheter–tissue contact, leading to inadequate lesion formation.^7^^,^^8^ Contributing factors may also include imprecise mapping and electrogram interpretation, albeit to a lesser extent. The importance of effective catheter–tissue contact has driven the development of ablation catheters equipped with real-time contact-force (CF) sensing capabilities, allowing direct quantification of the force applied at the catheter tip–tissue interface.^9^ This technology complements conventional surrogate indicators of contact—such as tactile feedback, electrogram attenuation, fluoroscopic visualization, impedance drop, and response to ablation—by providing a measurable and objective parameter.

To date, CF technology has been studied primarily in the setting of atrial fibrillation (AF) ablation.^10^, ^11^, ^12^, ^13^, ^14^ Although randomized trials demonstrated improvements in procedural efficacy, such as reduced procedure duration and fewer reconnections, they have not consistently shown improved freedom of AF. Despite widespread enthusiasm for CF technology in AF ablation, its application in SVT ablation remains underexplored, with only a limited number of small, nonrandomized studies evaluating its feasibility and clinical utility.^15^, ^16^, ^17^ Given that SVT ablation may particularly benefit from the improved catheter–tissue contact offered by CF guidance, this randomized controlled trial aimed to assess whether a CF-sensing approach is superior to conventional non-CF-guided ablation. The study specifically evaluated the number of radiofrequency (RF) applications required and compared the safety, procedural efficacy, and long-term outcomes between the 2 approaches.

## Methods

### Study design

The Contact-Force-Sensing-Based Radiofrequency Catheter Ablation in Paroxysmal Supraventricular Tachycardias study was a prospective, single-center, open-label, randomized controlled trial. The study protocol was approved by the medical research ethics committee (2017-394, NCT04078685) of the Erasmus Medical Center in Rotterdam, The Netherlands. Patient enrollment commenced in June 2018 and ended in March 2020. A written informed consent was obtained from all participants. The study was conducted according to the principles of the Declaration of Helsinki. The detailed design and methodology have been previously published.^18^

### Patient population

Patients referred for a standard electrophysiological study were prospectively enrolled if they presented with 1 or more of the following criteria: (1) pre-excitation on a 12-lead surface electrocardiogram (ECG), (2) palpitations with documented narrow-complex tachycardia, or (3) clinical features highly suggestive of paroxysmal SVT, such as sudden onset and termination of rapid, regular palpitations. Eligibility for randomization required confirmation of AVNRT or Wolff-Parkinson-White syndrome–AVRT (WPW-AVRT) during the electrophysiology study. The term WPW-AVRT describes patients with evidence of a manifest accessory pathway, with or without inducible AVRT, and those presenting with documented orthodromic AVRT involving a concealed bypass tract. Subject who met any of the following exclusion criteria were excluded from participating in this study: evidence of structural heart disease, myocardial ischemia, or pregnancy (or the inability to exclude it). Full inclusion and exclusion criteria have been previously published.^18^

### Randomization process

Patients were randomized in a 1:1 ratio to either catheter ablation using a CF-sensing ablation catheter (TactiCath Quartz, Abbott, Abbott Park, IL) or conventional ablation using a standard (non-CF-sensing, nonirrigated) ablation catheter (Celsius, Johnson & Johnson, New Brunswick, NJ, or AlCath FullCircle, Biotronik, Berlin, Germany). Randomization was conducted via a computer-generated program (ALEA) using block randomization (block size = 4). The process was overseen by personnel not involved in patient care or selection. Randomization occurred after the diagnostic portion of the procedure, upon notification by the treating electrophysiologist. The study followed an open-label design; neither patients nor electrophysiologists were blinded to the assigned treatment.

### Ablation strategy

For a standard diagnostic electrophysiological study, 3 short introducer sheaths (6F, 6F, and 8F) were typically inserted into the right femoral vein. A decapolar catheter was positioned in the coronary sinus, a quadripolar catheter in the right ventricle, and another quadripolar catheter at the His bundle position. 3-dimensional electroanatomic mapping was not performed. After placement, standard measurements were obtained, including basic conduction intervals, anterograde and retrograde Wenckebach points, and assessment for the presence of an AH jump and accessory pathways. SVT was induced using atrial extrastimuli or rapid atrial burst pacing, with or without the administration of isoprenaline. When AVNRT was diagnosed, the ablation target was the right-inferior extension of the slow pathway. The ablation catheter was positioned to produce a small atrial electrogram and a large ventricular electrogram, indicative of appropriate placement. RF ablation for slow pathway modification was delivered in a titrated fashion in a nonirrigated mode (10–55 W, 55°C, 1–2 mL/min, 60–90 seconds). The ablation goal was to elicit junctional beats and observe an impedance drop of 5–10 Ω. If necessary, additional ablation was performed in the proximal coronary sinus roof or from the left atrium to target the left inferior extension. For accessory pathway–mediated arrhythmias, RF energy (50–55 W, 55°C, 1–2 mL/min, 60–90 seconds) was applied at the site of the earliest ventricular activation during antegrade conduction or at the earliest retrograde atrial activation during orthodromic AVRT. If a distinct accessory pathway potential was visible, it was specifically targeted. The ablation goal was to eliminate accessory pathway conduction within 6 seconds of RF energy application. Irrigated catheters can be used in a “temperature-controlled mode” with the lowest possible irrigation mode (2 mL). A “temperature-controlled” mode can be achieved using irrigated catheters: with the EnSite system, this can be done by decoupling the generator from the irrigation control, with the Biosense system, the SmartAblate generator can be manually configured for the lowest irrigation flow compatible in the temperature-controlled mode. In cases requiring additional lesion formation or when initial ablation was unsuccessful, irrigated RF ablation (40 W, 43°C, 20 mL/min) could be used, but this was considered to be a protocol violation. Stable catheter–tissue contact (minimum of 10 seconds in duration) with a minimum CF exceeding 5–10 g (and an optimal CF within the range of 15–20 g) was targeted in the CF-sensing group. A long steerable sheath (Agilis, Abbott) was used for left-sided substrates and at operator discretion.

The ablation procedure was continued until the clinical tachycardia was no longer inducible and/or the conduction through an accessory pathway was terminated, provided that these clinical endpoints could be achieved with acceptable clinical risk–benefit ratios (eg, no signs of imminent complete atrioventricular [AV] block, no signs of pericardial effusion). Thereafter, the inducibility of the clinical tachycardia and/or conduction over an accessory pathway was reassessed throughout a waiting time of 30 minutes from the last RF application. Isoprenaline was used in case inducibility of the SVT was achieved with isoprenaline.

The electrophysiological study and ablation procedures were performed by 5 experienced operators, all registered electrophysiologists in The Netherlands. According to national requirements, registered electrophysiologists are required to perform a minimum of 375 ablation procedures over a 5-year period. At the study site (Erasmus MC), the average procedural volume per operator is approximately 150–175 ablation procedures per year. At the time of study initiation, the participating operators had between 7 and 20 years of experience in catheter ablation, each having performed more than 1000 procedures.

### Clinical follow-up

All patients underwent physical examination, 12-lead ECG, and echocardiography before the procedure and again the next day. Outpatient follow-ups occurred at 3 and 12 months after the procedure, including physical examinations and 12-lead ECGs. Additional visits were scheduled in case of symptom recurrence, with further evaluation by Holter monitoring (up to 3 weeks) and/or a trans-telephonic event recorder as needed. If recurrence was confirmed, a repeat electrophysiology study was allowed. Recurrence of clinical arrhythmia was defined as the reappearance of preablation symptoms and documentation of the same type of narrow-complex tachycardia or pre-excitation via 12-lead ECG, Holter monitor, or event recorder. Alternatively, recurrence was confirmed if the arrhythmia could be reinduced during a repeat electrophysiology study. Adverse events were assessed during the procedure, before discharge, and at the 3- and 12-month follow-up visits. Major adverse events included death, acute myocardial infarction/coronary artery damage, major bleeding, abdominal bleeding, cardiac tamponade or pericardial effusion requiring intervention (and/or prolonging the duration of hospitalization), ischemic cerebral event and other procedure-related embolic events, high-degree AV block requiring pacemaker implantation, atrial esophageal fistula, phrenic nerve palsy, and vascular access complications (requiring intervention and/or prolonging the duration of hospitalization, eg, arteriovenous fistula, or aneurysm). Minor adverse events included mild pericardial effusion, minor bleeding, and vascular access complications not requiring intervention and temporary high-degree AV block or novel first-degree AV block not requiring pacemaker implantation.

### Study end points

The primary outcome was the number of RF ablations during the ablation procedure. Secondary outcome parameters included overall duration of RF applications, fluoroscopy time, total procedural duration, adverse events, acute and long-term procedural success.

### Sample-size calculation

Full details have been published previously.^18^ Briefly, the sample size was based on our historical data, which showed mean ± standard deviation of 4.81 ± 3.21 RF applications per procedure. Owing to skewed distribution, these historical data were first transformed to natural log scale and subsequently used for sample-size calculation using Student *t* test for 2 independent means. To detect a 25% reduction in RF applications in the CF ablation group, with 80% power, a 2-sided alpha of 0.05, a 15% dropout rate, and a 95% acute success rate, the required total sample size was calculated to be 112 patients.

### Statistical analysis

Categorical variables are presented as frequencies and percentages and were tested using the χ^2^ or Fisher exact test as appropriate. Continuous variables are reported as mean ± standard deviation or median (interquartile range), depending on distribution, and compared using either the 2-sample Student *t* test or the Wilcoxon rank-sum test. Freedom from SVT recurrence was evaluated using Kaplan–Meier analysis, with between-group comparisons made using the log-rank test. Pearson correlation was used to examine the correlation between CF metrics and number of RF applications. *P* < .05 was considered statistically significant.

## Results

**Figure 1:**
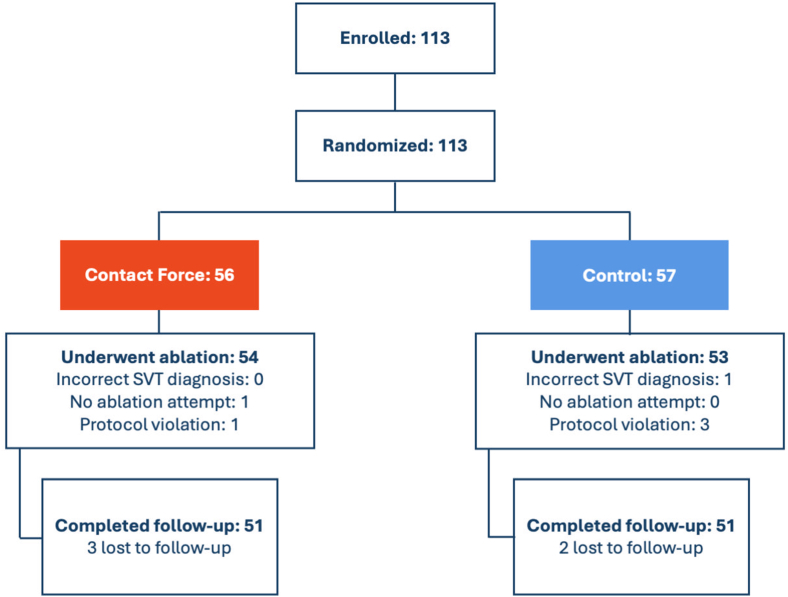
Study flow diagram. Flowchart of patient enrollment, randomization, and the populations included in various analysis. SVT = supraventricular tachycardia.

The patient flow through the 2 study arms is presented in Figure 1. A total of 113 patients were enrolled. 6 patients were excluded from the final analysis for the following reasons: incorrect diagnosis of SVT (n = 1), premature termination of the procedure before insertion of the ablation catheter owing to safety concerns (n = 1), use of a remote magnetic navigation system (n = 2), and replacement of a nonirrigated ablation catheter with an irrigated catheter before ablation in the control group (n = 2). The safety concern involved the presence of a para-Hisian accessory pathway.

Baseline demographic and clinical characteristics of the remaining 107 patients (54 in the CF arm and 53 in the control arm) are presented in Table 1. The mean patient age was 49.4 ± 14.7 years, and 47.7% were male. Most patients (91.6%) had normal left ventricular function. No significant baseline differences were observed between the 2 groups.

**Table 1:** Baseline table

Baseline characteristics	Total (N = 107)	CF (n = 54)	Control (n = 53)	*P* value
Age (y)	49.4 ± 14.7	47.7 ± 15.5	51.1 ± 13.7	.237
Men	47.7 (51)	50.0 (27)	45.2 (24)	.625
BMI	26.0 (22.8–29.7)	26.0 (23.3–29.1)	25.8 (22.5–30.4)	.728
Left ventricular systolic function				.706
Normal (55%)	91.6 (98)	92.6 (50)	90.6 (48)	
Fair (45%–54%)	8.4 (9)	7.4 (4)	9.4 (5)	
Left ventricular dilatation				1.0
Normal	100 (107)	100 (54)	100 (53)	
Symptoms	93.4 (99)	88.9 (48)	98.1 (52)	.123
Medication	56.3 (58)	57.7 (30)	54.9 (28)	.777
Beta-blocker	15.8 (17)	11.1 (6)	20.8 (11)	
Calcium antagonist	29.0 (31)	33.3 (18)	24.5 (13)	
Other medications	15.0 (16)	14.8 (8)	15.1 (8)	
Documentation of Tachycardia	96.3 (103)	98.1 (53)	96.2 (51)	.618
Ablation catheter				
TactiCath Quartz, Abbott	-	100 (54)	0 (0)	-
Celsius, Johnson & Johnson	-	0 (0)	100 (53)	-
AlCath FullCircle, Biotronik	-	0 (0)	0 (0)	-

Values are presented as percentages (numbers) or median (interquartile ranges).

BMI = body mass index; CF = contact force.

### Arrhythmia characteristics

Arrhythmia characteristics are presented in Table 2. During electrophysiological testing, AVNRT was diagnosed in most patients (n = 74; 69.2%), followed by manifest accessory pathway–mediated AVRT (n = 25; 23.4%) and concealed accessory pathway–mediated AVRT (n = 8; 7.4%). Most accessory pathways were located along the left free wall (n = 21; 63.6%). No patient exhibited both AVNRT and WPW-AVRT or had multiple accessory pathways. The distribution of arrhythmia subtypes was similar across both treatment arms.

**Table 2:** Substrate characteristics

Arrhythmia characteristics	CF	Control
AVNRT	34	40
Typical AVNRT	34	38
Atypical AVNRT	0	2
Slow-slow	-	1
Fast-slow	-	1
WPW-AVRT	20	13
Manifest	18	7
Concealed	2	6
Location		
Anteroseptal	0	0
Midseptal	2	0
Posteroseptal	4	3
Right free wall	2	1
Left free wall	12	9

AVNRT = atrioventricular nodal reentrant tachycardia; CF = contact force; WPW-AVRT = Wolff-Parkinson-White syndrome–atrioventricular reentrant tachycardia.

### CF metrics

In the CF arm, the mean force, force-time integral (FTI), and lesion size index (LSI) were 8.5 ± 6.9 g, 337.1 ± 367.7 gs, and 4.8 ± 1.9, respectively. Force and FTI values were significantly higher in patients with WPW-AVRT than those with AVNRT (force 13.7 g vs 5.3 g, *P* < .0001; FTI 570.8 vs 195.4 gs, *P* < .0001), whereas LSI values were similar (4.9 vs 4.7; *P* = .966). Among patients with WPW-AVRT, 90% had mean forces between 5 and 20 g, with 70% achieving the optimal 15–20 g range. In contrast, only 42% of patients with AVNRT were within the 5–20 g range, and none reached the optimal 15–20 g target.

### Procedural outcomes

**Figure 2:**
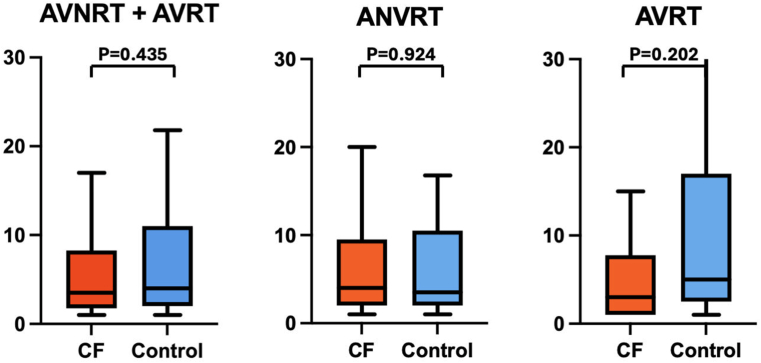
Number of RF applications. Left, middle, and right boxplots show the total number of RF applications in the CF approach (*orange*) and the conventional approach (*blue*) for all SVT cases, AVNRT only, and WPW-AVRT only, respectively. The main box represents median and interquartile range, and the *whiskers* represent 10th and 90th percentiles. AVNRT = atrioventricular nodal reentrant tachycardia; CF = contact-force; SVT = supraventricular tachycardia; WPW-AVRT = Wolff-Parkinson-White syndrome–atrioventricular reentrant tachycardia.

The number of RF applications across groups is presented in Figure 2. Median RF application numbers were 3.5 (1.8–8.3) in the CF arm and 4.0 (2.0–11.0) in the control arm (Wilcoxon rank-sum test *P* = .435). The results were similar when using the Student *t* test after transforming the number of RF applications using the natural logarithm, as applied in the power calculation (*P* = .312). Subgroup analysis demonstrated that the median application numbers did not differ significantly between treatment groups for the subgroups AVNRT and WPW-AVRT separately. In addition, no significant correlations were found between force, FTI, or LSI and the number of RF applications (r = 0.1–0.3; *P* > .05).

**Figure 3:**
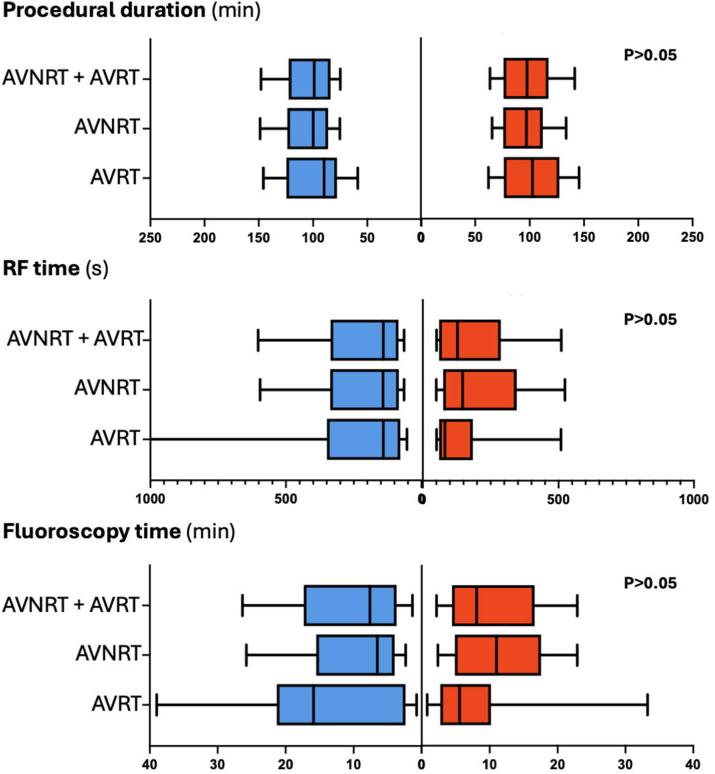
Procedural outcomes. Upper, middle, and lower boxplots display the total procedural duration, application duration, and fluoroscopy time, respectively, for the CF approach (*orange*) and the conventional approach (*blue*), across all SVT cases, AVNRT only, and WPW-AVRT only. The procedural duration includes the 30-minute waiting time. The main box represents median and interquartile range, and the *whiskers* represent 10th and 90th percentiles. AVNRT = atrioventricular nodal reentrant tachycardia; CF = contact-force; RF = radiofrequency; SVT = supraventricular tachycardia; WPW-AVRT = Wolff-Parkinson-White syndrome–atrioventricular reentrant tachycardia.

The total RF time was 128 seconds (60.0–287.3) in the CF arm and 141 seconds (84.5–336.0) in the control arm (*P* = .206), as presented in Figure 3. There were no significant differences in total procedural time or fluoroscopy time between the 2 arms in the overall cohort, nor within the AVNRT or WPW-AVRT subgroups separately (all *P* > .05).

### Acute and long-term outcomes

Acute procedural success was achieved in 98.1% of the patients in both arms. In each arm, 1 patient did not achieve acute success. In 1 patient in the CF arm, ablation near a midseptal accessory pathway resulted in transient loss of pre-excitation, but also in occurrence of nonconducting atrial beats. Although AV conduction recovered during the waiting period, no retrograde conduction was observed during ventricular pacing, prompting cessation of further ablation. In 1 patient in the control arm, ablation for slow-fast AVNRT initially failed to induce junctional beats; subsequent attempts higher in the septum induced a retrograde block, which was deemed too risky to continue despite inducible nonsustained AVNRT.

**Figure 4:**
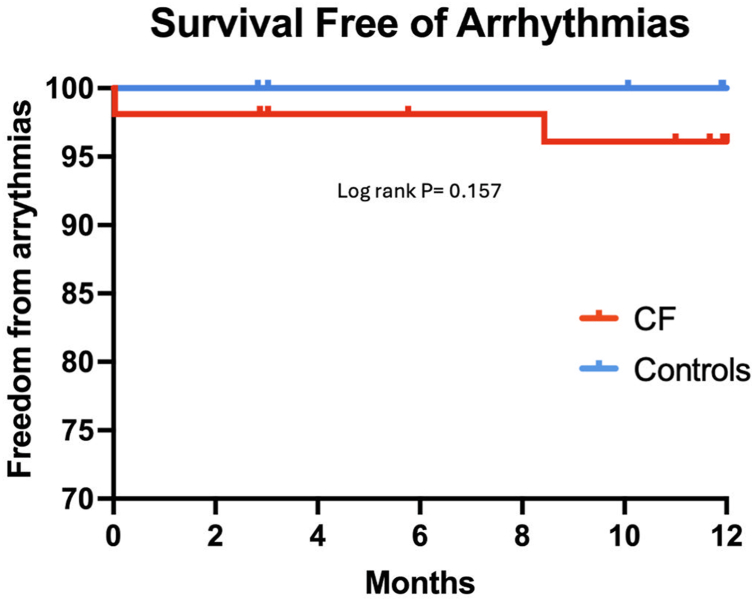
Long-term outcomes. Constructed Kaplan–Meier survival curves display the SVT-free survival rates after a single procedure in the CF arm (*orange*) and the conventional arm (*blue*). CF = contact-force; SVT = supraventricular tachycardia.

All patients completed the 3-month follow-up; 95.3% completed the 12-month follow-up, with 5 patients lost to follow-up. Single-procedure success at 12 months was 94.4% in the CF arm and 98.1% in the control arm (*P* = .618). Kaplan–Meier curves for SVT-free survival (log-rank *P* = .157) are presented in Figure 4. 2 patients in the CF arm experienced late recurrences and underwent successful redo procedures. 1 had recurrence of a manifest accessory pathway at the right posteroseptal region, ultimately ablated within the middle cardiac vein. The second patient had recurrent AVNRT ablated at the right-inferior extension near the posteroseptal region. In addition, 2 patients underwent repeat electrophysiological studies for palpitations, but no evidence of dual AV nodal physiology or inducible SVT was found.

### Safety outcomes

The incidence of adverse events was comparable between the 2 groups: 0% (0 of 54) in the CF arm and 5.7% (3 of 53) in the control arm (*P* = .118). 1 major adverse event occurred only in the control arm, involving a vascular access complication that prolonged hospitalization. The minor adverse events included deep vein thrombosis (n = 1) and persistent first-degree AV block (n = 1).

## Discussion

In this randomized controlled trial, the use of CF data during SVT ablation did not result in a significant reduction in the number of applications, regardless of arrhythmic substrate. Procedural efficacy and safety outcomes were similar between CF-guided and conventional ablation strategies. Both approaches demonstrated high acute and long-term success rates, with no statistically significant differences between the 2 arms.

### Clinical experience

CF technology was originally developed to optimize lesion formation in AF ablation by improving catheter–tissue contact and reducing complication rates.^9^ Drawing from this principle, we hypothesized that CF guidance could reduce the number of applications in SVT ablation by minimizing ineffective, poor-contact lesions. However, our findings did not support this hypothesis. Several factors may explain the lack of superiority of CF-guided ablation: first, experienced electrophysiologists may continue to rely on indirect markers of lesion efficacy—such as tactile feedback, electrogram morphology, and fluoroscopy—regardless of CF availability. Second, previous experience with CF may have subconsciously improved operator technique, even when CF data were not available in the conventional ablation group. Third, lesions may remain ineffective if the arrhythmic substrate is not clearly delineated, leading to a higher number of applications despite optimal CF. Fourth, additional applications are sometimes used to reinforce lesions after termination of tachycardia or the appearance of junctional ectopy. These factors suggest that the benefits of CF guidance may already be maximized under current standards of care. This is consistent with retrospective studies that have reported similar or even higher application counts in CF-guided SVT ablation than conventional methods.^15^, ^16^, ^17^

The high acute success and low recurrence rates observed in both arms reinforce the overall effectiveness of both ablation strategies, aligning with results from other high-volume centers.^2^^,^^5^ Furthermore, procedural metrics, such as fluoroscopy time, total RF time, and total procedural duration, were short and consistent with previous studies.^2^^,^^19^^,^^20^ These comparable outcomes also emphasize that it is clinically feasible to implement CF using irrigated catheters with low-flow settings, resulting in a virtual nonirrigated mode, which offers an alternative for operators facing challenging ablations. However, future improvement in outcomes is likely to be multifactorial and case specific, requiring advanced techniques such as alternative energy sources, high-resolution mapping, refined electrogram analysis (eg, unipolar, bipolar, and omnipolar signals), and enhanced catheter stability tools.

### CF metrics

The study protocol recommended a minimum of 10 seconds of stable contact with a force of >5–10 g and an optimal range of 15–20 g. In WPW-AVRT cases, 90% achieved forces within 5–20 g, and 70% fell within the optimal range. Although lower CF is typically used in WPW-AVRT than AF ablation, successful lesions in WPW-AVRT have been associated with higher forces.^8^^,^^16^ For AVNRT, reference CF values are lacking. In our cohort, forces were significantly lower, only 42% of cases achieved 5–15 g, and none reached 15–20 g. Given that AVNRT was the most common arrhythmia in the study, this may have influenced overall outcomes. However, it is important to note that catheter positioning on the slow pathway differs from that on the annulus for accessory pathways. In AVNRT, the catheter typically lies more parallel/oblique to the tissue surface, whereas, in WPW-AVRT, it tends to be more perpendicular. This parallel orientation is indeed associated with lower measured CFs in a porcine model.^21^ Nonetheless, both AVNRT and WPW-AVRT usually involve discrete, subendocardial targets not requiring transmural lesions. Importantly, higher forces did not correlate with fewer applications in our data. Future research with predefined CF targets per arrhythmia type may clarify the role of CF in SVT ablation. In addition, integrating advanced metrics such as FTI may promote more uniform lesion formation and potentially reduce application counts.

### Safety outcomes

The safety profile of CF-guided ablation was comparable with that of conventional approach, with low overall adverse event rates in both arms. Although excessive CF is theoretically linked to complications such as steam pops or cardiac perforation,^22^^,^^23^ these events are rare and likely require larger sample sizes to detect significant differences. The adverse events observed in this study involved vascular access issues or AV conduction disturbances, which are unlikely to be influenced by the ablation strategy.

### Economic evaluation

At present, there is a growing need to make assessments of the value of health care interventions, balancing costs with health outcomes. An important consideration of our findings relates to the economic implications of CF-sensing technology. Although exact list prices cannot be disclosed owing to regulatory and institutional policies, the CF catheter used in this study was associated with an additional cost of approximately €1374 per procedure compared with the conventional non–CF catheter. Although device costs represent a substantial component of procedural expenditure, overall cost-effectiveness is determined by a broader range of factors, including procedural efficiency, complication rates, and the need for repeat interventions. Previously, Mansour et al^24^ have suggested that CF-guided ablation in patients with AF may reduce overall health care costs, primarily driven by a decrease in arrhythmia recurrence and repeat procedures. However, in the present study, no differences were observed between groups with respect to acute or long-term success, safety outcomes, or procedural parameters. In the absence of reductions in these outcomes, the higher upfront cost of CF technology is unlikely to be offset by downstream savings. Therefore, from an economic perspective, our findings do not support a cost-effectiveness advantage of CF-guided ablation in this specific SVT population.

### Limitations

This study has several limitations. All procedures were performed by experienced operators familiar with both SVT ablation and CF technology. Thus, findings may not be generalizable to less experienced users, who might derive greater benefit from CF guidance. In the CF arm, target forces were not consistently achieved, particularly in AVNRT cases. Moreover, current CF thresholds are based on AF data and may not directly translate to SVT substrates. The study included various subtypes of WPW-AVRT and AVNRT, but the number of patients per subgroup was limited, precluding robust subgroup analyses. Although no significant differences were observed, small sample sizes may have masked clinically relevant differences. Future studies focusing on specific arrhythmic substrates may help identify differential effects of CF guidance.

## Conclusion

In this first randomized controlled trial investigating CF-guided ablation in SVT, CF guidance did not significantly reduce the number of RF applications and was not superior to conventional ablation in terms of procedural efficacy or safety. The high success rates in both arms suggest a procedural performance ceiling that may not be improved by CF alone. Further gains are likely to require individualized approaches based on substrate characteristics, procedural technique, and adjunctive technologies.

## Disclosures

S.C.Y. has received honoraria from Medtronic, Boston Scientific, Biotronik, Acutus Medical, and Sanofi. Furthermore, he has received institutional research grants from Medtronic, Biotronik, Boston Scientific, and Johnson & Johnson. T.S.T. reports educational activity and an advisory relationship with Biotronik, and until 2022, he was in educational, advisory, and product development relationships with Ablacon Inc, Abbott, Acutus Medical, Biosense Webster, and Stereotaxis. S.A.W. has received honoraria from Biotronik and Daiichi Sankyo. The other authors have nothing to declare.
